# Economic evaluation of dapagliflozin added to standard therapy in chronic heart failure in Argentina: a cost-utility analysis

**DOI:** 10.1016/j.lana.2026.101571

**Published:** 2026-07-15

**Authors:** Javier Santiago Araujo, Francisco Moioni, Diego Sebastián Novielli, Germán Andrés Jelusic, Sofía Inés Lucini, Jeremías Augusto Zabalo, José Alberto Spala, María Celeste Piervincenzi

**Affiliations:** Hospital Cuenca Alta Néstor Kirchner, Provincia de Buenos Aires, Argentina

**Keywords:** Cost-utility analysis, Cost-effectiveness, Dapagliflozin, Heart failure, Markov model, Quality-adjusted life years (QALYs), Incremental cost-effectiveness ratio (ICER), Argentina

## Abstract

**Background:**

Heart failure is associated with high morbidity, mortality, and substantial resource use. Dapagliflozin improves outcomes in heart failure, but local economic evidence for Argentina is limited. We conducted a cost-utility analysis of dapagliflozin added to standard therapy for chronic heart failure in Argentina.

**Methods:**

We conducted a cost–utility analysis from the Argentine public health system perspective using two trial-based Markov models (DAPA-HF and DELIVER populations), monthly cycles, lifetime horizon, and 3% annual discounting for costs and outcomes. Inputs were derived from published trial data and Argentine sources where available. We estimated costs, quality-adjusted life-years (QALYs), and incremental cost-effectiveness ratios (ICERs), and performed deterministic and probabilistic sensitivity analyses.

**Findings:**

In the DAPA-HF model, dapagliflozin increased QALYs by 0.76 with an incremental cost of US$5069, yielding an ICER of US$6672 per QALY. In the DELIVER model, dapagliflozin increased QALYs by 0.44 with an incremental cost of US$4542, yielding an ICER of US$10,371 per QALY. At a willingness-to-pay threshold of 1 gross domestic product (GDP) per capita, the probability of cost-effectiveness was 97% (DAPA-HF) and 73% (DELIVER).

**Interpretation:**

Dapagliflozin added to standard therapy is likely to be cost-effective in Argentina, with greater robustness in heart failure with reduced ejection fraction. In preserved or mildly reduced ejection fraction, cost-effectiveness is compatible with a 1 GDP per capita threshold but more sensitive to key assumptions; price negotiation could improve value for money.

**Funding:**

None.

**Research in context**

**Evidence before this study**

We searched PubMed, Embase, and regional databases for economic evaluations of sodium–glucose co-transporter 2 (SGLT2) inhibitors in heart failure, from database inception to December 2025, without language restrictions. Search terms included combinations of “dapagliflozin”, “heart failure”, “cost-effectiveness”, “economic evaluation”, and “cost–utility analysis”. We also screened reference lists of relevant articles and reviews. Available evidence consists mainly of cost-effectiveness analyses conducted in high-income settings and a limited number of studies from middle-income countries. Systematic reviews suggest that SGLT2 inhibitors are generally cost-effective in heart failure, particularly in reduced ejection fraction populations, although results are more heterogeneous in preserved or mildly reduced ejection fraction. However, there is a scarcity of locally generated economic evidence in Latin America, and no published cost-effectiveness analyses specific to Argentina were identified. The available studies are subject to limitations related to transferability, given differences in costs, health system organization, and epidemiology.

**Added value of this study**

This study provides, to our knowledge, the first cost–utility analysis of dapagliflozin in heart failure conducted in Argentina, using locally adapted inputs for costs, healthcare resource use, and epidemiological context. By developing two separate models based on populations from major randomized clinical trials, this analysis captures differences across the spectrum of ejection fraction. The study incorporates local cost data derived from real-world practice in a public hospital and reflects the Argentine healthcare system perspective. These results contribute context-specific evidence to inform decision-making in a setting where economic evaluations are scarce and access to high-cost therapies remains limited.

**Implications of all the available evidence**

The available evidence suggests that dapagliflozin is likely to be a cost-effective intervention for heart failure, particularly in reduced ejection fraction, across different settings. Our findings reinforce this conclusion in the Argentine context and highlight the potential role of price negotiation and procurement strategies in improving value for money, especially in populations with preserved or mildly reduced ejection fraction. In the absence of structured reimbursement or cost-containment mechanisms in Argentina, this study provides relevant evidence to support coverage decisions and policy development aimed at improving equitable access. Future research should incorporate real-world data and explore subgroup and equity considerations to better inform implementation in diverse health system settings.

## Introduction

Heart failure (HF) is a progressive and highly prevalent condition associated with impaired quality of life, recurrent hospitalizations, and substantial mortality. In Argentina, as in other Latin American countries, HF is a frequent cause of hospitalization among older adults and imposes a considerable burden on the public health system, driven mainly by acute episodes requiring inpatient care.^1^ In this context, sodium–glucose co-transporter 2 (SGLT2) inhibitors have emerged as an effective therapeutic option, demonstrating reductions in the risk of hospitalization and cardiovascular death when added to standard therapy. SGLT2 inhibitors are a class of glucose-lowering agents initially developed for the treatment of type 2 diabetes mellitus. Subsequently, large randomized clinical trials demonstrated important cardiovascular and renal benefits independent of glycemic status, leading to expanded indications that include chronic heart failure and chronic kidney disease. Among these agents, dapagliflozin has consistently reduced heart failure hospitalization and cardiovascular mortality when added to standard therapy. Dapagliflozin showed consistent clinical benefits in the randomized DAPA-HF^2^ and DELIVER^3^ trials, leading to its incorporation into national and international guidelines as part of the pharmacological management of HF. Nevertheless, the adoption of new therapies in public health systems requires assessment not only of clinical efficacy but also of their economic impact in heterogeneous regional settings. Although international analyses suggest that dapagliflozin may be cost-effective, economic evidence from the region remains limited, underscoring the need for evaluations adapted to local contexts.^4^^,^^5^ Against this background, the objective of this study was to conduct a cost-utility economic evaluation of dapagliflozin added to standard therapy in patients with chronic HF, from the perspective of the Argentine healthcare system, to estimate costs, quality-adjusted life-years (QALYs), and the incremental cost-effectiveness ratio (ICER).

## Methods

### Study design and strategies evaluated

The cost-utility economic analysis was conducted from the perspective of the Argentine public health system, considering exclusively direct medical costs. Given the chronic and progressive nature of HF, the base-case analysis adopted a lifetime horizon to capture long-term differences in survival, recurrent clinical events, costs, and quality-adjusted survival between strategies. In addition, alternative time horizons of 1, 5, and 10 years were explored in scenario analyses to assess the sensitivity of results to the duration over which treatment benefits were accrued. Two independent state-transition models were developed, based on the major clinical trials of dapagliflozin in HF: (i) a model structured on the DAPA-HF trial, representative of patients with reduced ejection fraction, and (ii) a model derived from the DELIVER trial, applicable to a population with preserved or mildly reduced ejection fraction. In both cases, two therapeutic strategies were compared: standard HF therapy and dapagliflozin 10 mg daily added to standard therapy. Standard therapy was assumed to be consistent with national and international guideline recommendations for the pharmacological management of HF.^6^, ^7^, ^8^ The primary outcome was the ICER, expressed as cost per QALY gained with dapagliflozin compared with conventional treatment. Life-years gained were estimated as a secondary outcome. Results were interpreted against a willingness-to-pay range of 0.5–1 gross domestic product (GDP) per capita, proposed based on health expenditure growth and life expectancy, as described by Pichon-Riviere et al.^9^ Considering Argentina's most recently available GDP per capita estimate, USD 13,858,^10^ the corresponding threshold range was set at USD 6929 to USD 13,858 per QALY gained. To ensure methodological rigor and reporting quality, the analysis was conducted in accordance with the Consolidated Health Economic Evaluation Reporting Standards (CHEERS) 2022 guidelines^11^ (Supplementary Table S1). A formal study protocol was not prospectively registered, as this was a model-based economic evaluation using secondary data sources.

### Population

Our hypothetical cohort included 1000 patients in each of the two models, with demographic and clinical characteristics aligned with the populations studied in the clinical trials. In the model based on DAPA-HF, patients entered the initial cycle at a mean age of 66 years, corresponding to the mean age reported in the trial, with a diagnosis of chronic HF with reduced ejection fraction. DAPA-HF was a randomized, double-blind, placebo-controlled trial that enrolled adults with symptomatic chronic HF (NYHA class II–IV) and an ejection fraction ≤40%, receiving optimized standard pharmacological therapy. Participants were assigned to dapagliflozin 10 mg daily or placebo, in addition to standard therapy, with a median follow-up of 18.2 months. In parallel, a second economic model was developed based on the DELIVER trial, in which patients entered at a mean age of 71 years, corresponding to the mean age reported in the trial, with a diagnosis of HF with preserved or mildly reduced ejection fraction. DELIVER was a multicenter, randomized, double-blind, placebo-controlled study that enrolled symptomatic adults (NYHA class II–IV) with an ejection fraction >40%, who were assigned to receive dapagliflozin or placebo in addition to standard therapy, with a median follow-up of 2.3 years. Full baseline characteristics of participants in both trials are available in the original publications. This economic modeling study did not require review by an ethics committee, as it exclusively used publicly available secondary data from clinical trials and previously published sources.

### Model description

**Fig. 1:**
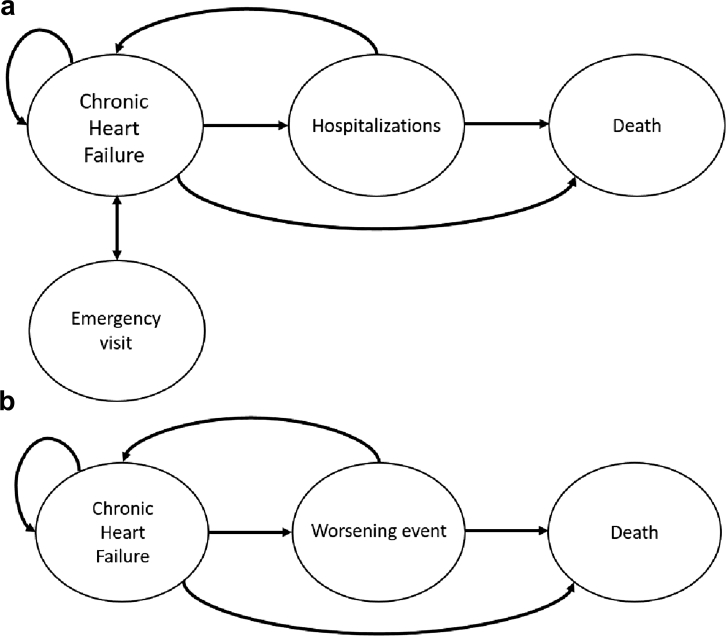
a. Markov model structure for DAPA-HF. b. Markov model structure for DELIVER.

Two state-transition Markov models were developed, conceptually adapted from the framework proposed by Bhatt et al.,^12^ to represent the burden and clinical course of chronic HF using monthly cycles. Differences in the availability and type of information reported in the clinical trials motivated the construction of two structurally distinct implementations, while maintaining a common conceptual analytical framework. In the model based on DAPA-HF, four mutually exclusive health states were defined: stable HF, HF hospitalization, HF urgent visit, and death from any cause (absorbing state) (Fig. 1a). All patients entered the model in the stable state and, in each monthly cycle, could remain in that state, experience a hospitalization or an urgent visit, or die. The inclusion of a specific urgent visit state was driven by the fact that the DAPA-HF trial reported information only for the first urgent visit, whereas total events (first and recurrent) were available for hospitalizations from pre-specified trial analyses.^13^ The hospitalization and urgent visit states represent acute events modeled as transient one-cycle states, after which patients return to the stable state or die. In the model based on DELIVER, a three-state mutually exclusive structure was used: stable HF, worsening HF event, and death from any cause (absorbing state) (Fig. 1b). The worsening HF state was defined as a composite outcome including HF hospitalizations and urgent visits, and was parameterized using total event rates (first and recurrent) available from pre-specified trial analyses.^14^ This information allowed the combined incidence of acute episodes to be modeled directly as a transition from the stable state. In each cycle, costs, life-years, and QALYs were accrued. The model was implemented in Microsoft Excel.

### Clinical parameters

#### All-cause mortality

All-cause and cardiovascular mortality rates for the control arm were obtained from the DAPA-HF and DELIVER trials. In DAPA-HF, all-cause and cardiovascular mortality rates were 9.5 and 7.9 events per 100 patient-years, respectively, while in DELIVER they were 7.6 and 3.8 events per 100 patient-years. To extrapolate beyond the trial follow-up period, the observed rates were compared with age-specific expected mortality in the Argentine population using national life tables,^15^ applying an approach based on the declining exponential approximation of life expectancy (DEALE). It was assumed that the excess mortality associated with HF observed in the trials remains constant over time, and the ratio between trial-observed mortality and population mortality was used to adjust age-specific baseline mortality. This procedure allowed the generation of age-specific annual mortality rates for the placebo arm starting from the initial age at model entry. Subsequently, total mortality was disaggregated into cardiovascular and non-cardiovascular mortality using the proportions reported in each trial. For the dapagliflozin arm, age-specific mortality rates were obtained by applying the reported hazard ratios for all-cause and cardiovascular mortality. Non-cardiovascular mortality was derived by difference, ensuring internal consistency among the components of total mortality. Finally, all age-specific annual rates were converted into monthly probabilities to inform transitions within the Markov model, assuming a constant hazard within each monthly cycle.

#### Worsening event

Transition probabilities associated with clinical events were estimated from the annual rates reported in the clinical trials and their pre-specified analyses.^13^^,^^14^ In the model based on DAPA-HF, the annual rate of the total events endpoint combining HF hospitalizations (first and recurrent) and cardiovascular death was used, estimated at 21.6 events per 100 patient-years.^13^ Because hospitalizations occurring on the day of death were not counted in this analysis, the annual rate of total hospitalizations was estimated as the difference between the composite endpoint rate and the reported cardiovascular mortality rate; this yielded 13.7 events per 100 patient-years.^13^ The rate of HF urgent visits was obtained from the DAPA-HF study and corresponded only to first events (0.7 events per 100 patient-years); therefore, a constant rate for this event was assumed over the model horizon. In the model based on DELIVER, annual rates of the total events endpoint (first and recurrent) combining HF hospitalizations and urgent visits (15.3 events per 100 patient-years), as well as the reported cardiovascular mortality rate, were used.^14^ Consistent with the analysis reported by Jhund et al., and considering that hospitalizations occurring on the day of death were not counted, total event rates specific to hospitalizations and urgent visits were derived for incorporation into the model (11.5 events per 100 patient-years).^14^ The hospitalization-to–urgent visit proportion was used to allocate costs and utilities and to apply in-hospital mortality exclusively to hospitalizations. In both model implementations, annual rates were converted into monthly probabilities using exponential conversion, assuming a constant risk within each monthly cycle. In-hospital mortality due to HF was estimated from the Argentine Registry of Acute Heart Failure (ARGEN-IC)^16^ and applied to hospitalized patients in each cycle. In the DELIVER model, in-hospital mortality was applied to the fraction of worsening HF events attributable to hospitalization, using the hospitalization-to–urgent visit proportion reported in the studies.^14^ Hazard ratios for dapagliflozin for the different outcomes were obtained from the original publications and applied to the control arm.^2^^,^^3^^,^^13^^,^^14^

### Quality of life

Given the lack of local utility estimates for HF in Argentina, utility values from the international literature were used. Baseline utility for the stable health state was set at 0.88 for the DAPA-HF model and 0.87 for the DELIVER model, in accordance with the estimates reported by Yang et al.,^17^ derived from a pooled analysis of DAPA-HF and DELIVER with mapping to EQ-5D. Although these values correspond to different populations, they were assumed to be representative of the Argentine population due to the absence of specific local data. Disutilities associated with acute events were incorporated based on Davis et al.^18^: a one-time disutility of 0.02 was assigned to each HF hospitalization and a disutility of 0.06 to each urgent care event. These values were applied directly without regional adjustment, as no corresponding estimates are available for the Argentine HF population. QALYs were calculated by accumulating event-adjusted utilities over model cycles and were discounted at an annual rate of 3%, in line with standard methodological recommendations for economic evaluations.^19^

### Costs

The analysis was conducted from the perspective of the Argentine public health system and included exclusively direct medical costs. All costs were expressed in 2025 Argentine pesos and converted to U.S. dollars (USD) using the official exchange rate of the Central Bank of the Argentine Republic as of December 1, 2025 (1 USD = ARS 1423.76). The price date used for resources, procedures, and medications was December 1, 2025, and when historical values required updating, inflation adjustments were applied using the Consumer Price Index of the National Institute of Statistics and Censuses up to that date. The cost of HF hospitalization was estimated based on the values reported by Augustovsky et al.,^20^ which were updated for inflation to December 1, 2025 to reflect contemporary costs in the Argentine context. This cost was applied to each modeled hospitalization episode. For HF urgent visit and the monthly cost of chronic HF management, in the absence of Argentina-specific estimates, the study by Naves et al.^21^ was used as a reference framework to identify relevant resources and care processes. These components were adapted to the local context according to routine clinical practice, through review and validation by cardiologists with clinical activity at the Hospital de Cuenca Alta “Néstor Kirchner.” The identified resources were subsequently valued using the Hospital's actual procurement costs, with the aim of reflecting resource consumption within the local public health system (Supplementary Tables S2 and S3). Given the variability in available brands and formulations, medication costs were estimated using the median market prices in Argentina for each active ingredient included in standard HF therapy and for dapagliflozin. Costs were adjusted according to dosing recommendations from national and international guidelines and to adherence levels reported in HF clinical trials (Supplementary Table S4). Both costs and health outcomes were discounted at an annual rate of 3%.^19^ Inputs for both models are presented in Tables 1 and 2.

**Table 1:** Model inputs for the DAPA-HF model.

Parameter	Base value	Lower bound	Upper bound	Distribution	Source
Monthly probability of HF hospitalization (first and recurrent)—placebo	0.0114	0.0100	0.0130	Beta	^13^
Monthly probability of CV death—placebo	0.0066	0.0045	0.0088	Beta	^2^
Proportion of CV death/total death—placebo	0.8298	–	–	–	^2^
Monthly probability of HF urgent visit—placebo	0.0006	0.0003	0.0010	Beta	^2^
HR dapagliflozin: HF hospitalization (total)	0.71	0.61	0.82	Log-normal	^13^
HR dapagliflozin: CV death	0.82	0.69	0.98	Log-normal	^2^
HR dapagliflozin: non-CV death	0.83	0.71	0.97	Log-normal	^2^
HR dapagliflozin: HF urgent visit	0.43	0.20	0.90	Log-normal	^2^
In-hospital mortality probability for HF	0.0790	0.0600	0.1000	Beta	^16^
Cost of HF hospitalization (USD)	3937.61	3150.09	4725.13	Gamma	^20^
Cost of HF urgent visit (USD)	101.93	81.54	122.32	Gamma	Supplementary Table S2
Monthly cost of chronic HF management (USD)	169.39	135.51	203.26	Gamma	Supplementary Table S3
Monthly cost of dapagliflozin (USD)	47.33	37.87	56.80	Gamma	Supplementary Table S4
Baseline utility in HF	0.88	0.75	0.95	Beta	^17^
Disutility per HF hospitalization	0.0200	0.0000	0.0400	Beta	^18^
Disutility per HF urgent visit	0.0600	0.0000	0.1000	Beta	^18^

Abbreviations: CV, cardiovascular; HF, heart failure; HR, hazard ratio; USD, United States dollars.

**Table 2:** Model inputs for the DELIVER model.

Parameter	Base value	Lower bound	Upper bound	Distribution	Source
Monthly probability of worsening HF event—placebo	0.0095	0.0073	0.0118	Beta	^14^
Monthly probability of CV death—placebo	0.0032	0.0019	0.0045	Beta	^3^
Proportion of CV death/total death—placebo	0.4962	–	–	–	^3^
HR dapagliflozin: worsening HF event	0.72	0.65	0.81	Log-normal	^14^
HR dapagliflozin: CV death	0.88	0.74	1.05	Log-normal	^3^
HR dapagliflozin: non-CV death	0.94	0.83	1.07	Log-normal	^3^
In-hospital mortality probability for HF	0.0702	0.0615	0.0965	Beta	^16^
Proportion of hospitalization/urgent visit events	0.8882	–	–	–	^14^
Cost of HF hospitalization (USD)	3937.61	3150.09	4725.13	Gamma	^20^
Cost of HF urgent visit (USD)	101.93	81.54	122.32	Gamma	Supplementary Table S2
Monthly cost of chronic HF management (USD)	117.50	94.00	141.00	Gamma	Supplementary Table S3
Monthly cost of dapagliflozin (USD)	47.33	37.87	56.80	Gamma	Supplementary Table S4
Baseline utility in HF	0.87	0.75	0.95	Beta	^17^
Disutility per HF hospitalization	0.0200	0.0000	0.0400	Beta	^18^
Disutility per HF urgent visit	0.0600	0.0000	0.1000	Beta	^18^

Abbreviations: CV, cardiovascular; HF, heart failure; HR, hazard ratio; USD, United States dollars.

### Statistical analysis

#### Sensitivity analysis and scenarios

Deterministic and probabilistic sensitivity analyses were conducted to assess the impact of uncertainty associated with the main clinical, epidemiological, and cost parameters included in the model. In the one-way deterministic sensitivity analysis, each parameter was varied individually within its lower and upper bounds. When the literature reported confidence intervals (95% CI), these values were used; when specific ranges were not available, variations of ±10% were assumed for probabilities and utilities and ±20% for costs. The effect of these variations on the ICER was displayed using tornado diagrams, in which the length of each bar reflects the magnitude of the impact of the corresponding parameter, ordered from greatest to least influence. The probabilistic sensitivity analysis was performed using 5000 model iterations through a second-order Monte Carlo simulation to capture parameter uncertainty, whereby input parameters were simultaneously sampled from predefined probability distributions. Beta distributions were used for probabilities and utilities, gamma distributions for costs, and log-normal distributions for hazard ratios associated with dapagliflozin treatment. Results of the probabilistic analysis were presented using cost-effectiveness plane scatter plots and cost-effectiveness acceptability curves. In addition, structural scenario analyses were conducted to evaluate the robustness of results to changes in key model assumptions. Alternative scenarios explored variations in the discount rate (1%, 5%, and 10%) and reductions in the time horizon from lifetime to 1, 5, and 10 years. A scenario with more conservative baseline utilities for the stable health state was considered to reflect potentially lower quality of life in real-world clinical practice and to capture uncertainty related to the use of international estimates.^22^ Finally, scenarios assessing reductions in the price of dapagliflozin were analyzed, applying decreases of 10%, 25%, and up to 50% relative to the base-case value, consistent with potential public procurement conditions and price negotiations within the Argentine healthcare system. In addition, a structural scenario analysis was conducted to explore uncertainty in long-term mortality extrapolation, assuming progressive attenuation of the heart failure–related excess mortality over age-specific Argentine background mortality in older age groups.^23^

### Validation

The internal consistency of the model and the plausibility of its assumptions were assessed through a process of conceptual and technical validation. The model structure, state transitions, and coherence of generated results were reviewed iteratively, including discussions with a multidisciplinary team comprising professionals in cardiology, epidemiology, social work, pharmacy, and health economics, with the aim of ensuring that model behavior adequately reflected the natural history of HF and the available clinical evidence. External validity was examined by comparing model projections with results reported in previous economic evaluations and clinical studies assessing the cost-effectiveness of dapagliflozin in HF.^4^^,^^5^ In addition, the epidemiological plausibility of the model was assessed using data from the Argentine OFFICE–IC–AR registry, a prospective real-world cohort of patients with chronic HF.^24^ The DAPA-HF population was comparable to the reduced ejection fraction subgroup of OFFICE–IC–AR in terms of age (66.3 vs 64.6 years) and left ventricular ejection fraction (31% vs 29.6%). Likewise, the DELIVER population showed similar characteristics to the mildly reduced and preserved ejection fraction subgroups of the registry (mean age 71.7 vs 66.7–70.5 years; mean ejection fraction 54% vs 44.8–58.4%). Annual cardiovascular mortality in OFFICE–IC–AR ranged from 6.5% to 8.4%, consistent with mortality estimates generated by the model. However, heart failure hospitalization rates reported in the registry (8.4%–10.1% annually) were lower than those projected by the trial-based models. Overall, these findings supported the epidemiological plausibility and external validity of the model in the Argentine setting. The choice of probability distributions used in the probabilistic sensitivity analysis was also assessed for methodological consistency. Beta distributions were assigned to probabilities and utilities because these parameters are bounded between 0 and 1, gamma distributions were used for cost variables given their non-negative and right-skewed nature, and log-normal distributions were applied to hazard ratios to preserve positivity and multiplicative treatment effects. These selections are consistent with standard recommendations for probabilistic sensitivity analysis in health economic modeling.

### Role of the funding source

This study received no external funding. No external entity had any role in study design, data collection, data analysis, data interpretation, writing of the report, or the decision to submit for publication. All authors had full access to the data used in the study and accept responsibility for the decision to submit.

## Results

### Base case

In the base-case analysis based on DAPA-HF, standard therapy plus dapagliflozin was associated with an increase of 0.76 QALYs and 0.86 life-years, at an incremental cost of USD 5069.22, resulting in an ICER of USD 6671.61 per QALY gained compared with standard therapy alone (Table 3). In the DELIVER-based model, standard therapy plus dapagliflozin was associated with gains of 0.44 QALYs and 0.50 life-years, at an incremental cost of USD 4542.39 compared with standard therapy alone. The resulting ICER was USD 10,371.29 per QALY gained (Table 4).

**Table 3:** Base-case results (DAPA-HF model).

Strategy	Costs (USD)	QALYs	Δ Costs (USD)	Δ QALYs	Life-years	Δ Life-years	ICER (USD/QALY)
Standard therapy	15,401.84	5.32	–	–	6.05	–	–
Standard therapy + dapagliflozin	20,471.06	6.08	5069.22	0.76	6.91	0.86	6671.61

Abbreviations: ICER, incremental cost-effectiveness ratio; QALY, quality-adjusted life-year; USD, United States dollars.

**Table 4:** Base-case results (DELIVER model).

Strategy	Costs (USD)	QALYs	Δ Costs (USD)	Δ QALYs	Life-years	Δ Life-years	ICER (USD/QALY)
Standard therapy	13,452.85	6.50	–	–	7.48	–	–
Standard therapy + dapagliflozin	17,995.23	6.94	4542.39	0.44	7.98	0.50	10,371.29

Abbreviations: ICER, incremental cost-effectiveness ratio; QALY, quality-adjusted life-year; USD, United States dollars.

### Sensitivity analysis

**Fig. 2:**
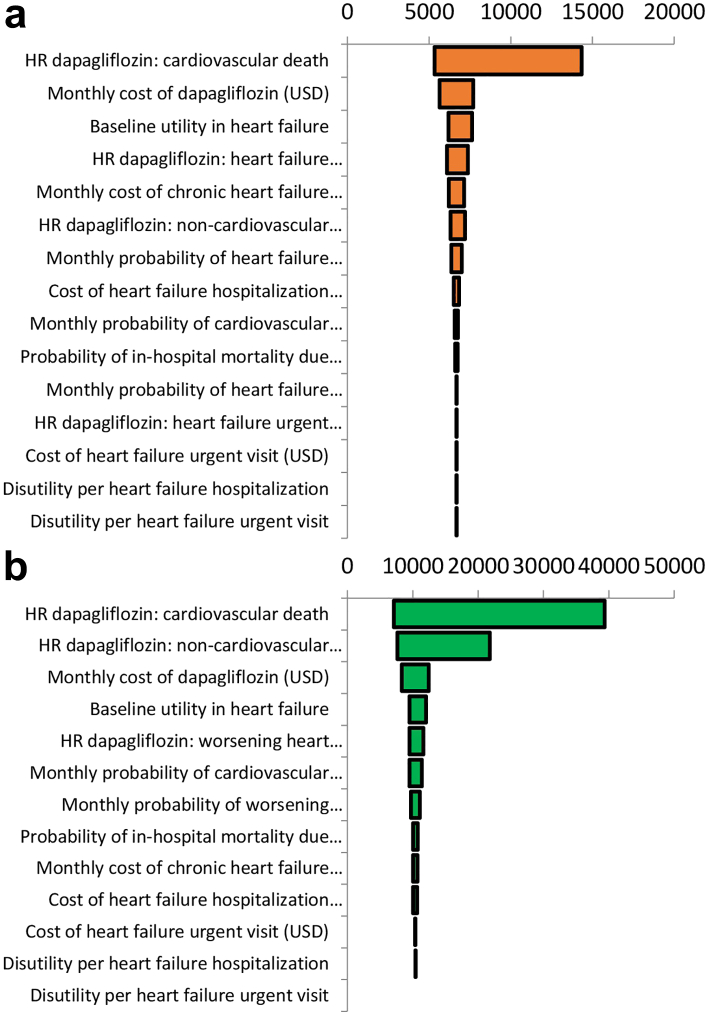
Tornado diagrams showing the individual impact of variations in key model parameters on the ICER of dapagliflozin vs standard therapy in the DAPA-HF population (a) and the DELIVER population (b). Each bar represents the range of ICER values obtained by replacing the base-case value of each parameter with its lower and upper bounds, while holding all other model assumptions constant. Parameters are ordered according to their relative influence on the ICER. Abbreviations: CV, cardiovascular; HF, heart failure; HR, hazard ratio; ICER, incremental cost-effectiveness ratio; USD, United States dollars.

For the DAPA-HF–based model, the one-way deterministic sensitivity analysis showed that the ICER was most sensitive to parameters related to the treatment effect on cardiovascular mortality, followed by the monthly cost of dapagliflozin and the baseline utility of the chronic HF health state (Fig. 2a). For the DELIVER-based model, the deterministic sensitivity analysis indicated that the ICER was primarily sensitive to parameters associated with the effect of dapagliflozin on mortality, particularly the hazard ratio for cardiovascular death, which was the single most influential determinant of the results (Fig. 2b).

**Fig. 3:**
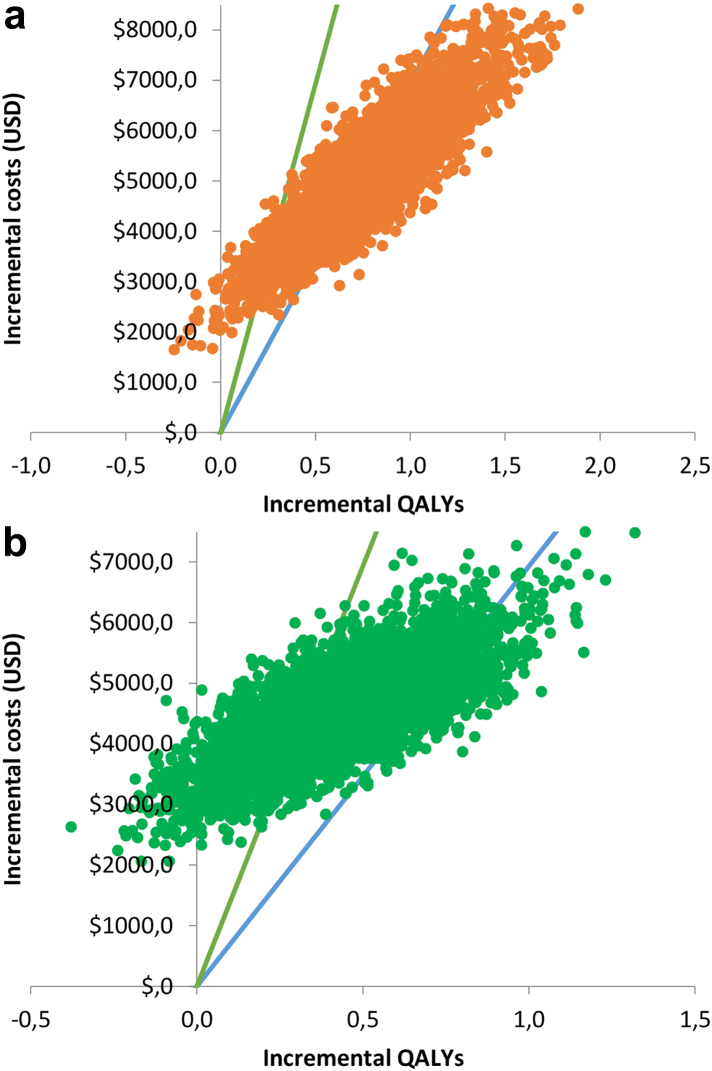
Cost-effectiveness planes from the probabilistic sensitivity analysis for DAPA-HF (a) and DELIVER (b). Each point represents one iteration of the probabilistic sensitivity analysis, showing the incremental costs (USD) and incremental QALYs of dapagliflozin compared with standard therapy. The diagonal lines correspond to willingness-to-pay thresholds equivalent to 0.5 times GDP per capita (blue line) and 1 GDP per capita (green line), at USD 6929 and USD 13,858 per QALY, respectively. The dispersion of points reflects the joint uncertainty of the model parameters. Abbreviations: GDP, gross domestic product; QALY, quality-adjusted life-year; USD, United States dollars.

In the probabilistic sensitivity analysis for the DAPA-HF model, 56% of iterations were cost-effective using a conservative threshold equivalent to 0.5 times GDP per capita (≤ USD 6929 per QALY). When a threshold of 1 GDP per capita (≤ USD 13,858 per QALY) was applied, the probability of cost-effectiveness increased to 97%, indicating a high likelihood that the dapagliflozin strategy is cost-effective under less restrictive willingness-to-pay assumptions (Fig. 3a). In the probabilistic sensitivity analysis for the DELIVER model, 9% of iterations were cost-effective using a conservative threshold equivalent to 0.5 times GDP per capita (≤ USD 6929 per QALY). When a threshold of 1 GDP per capita (≤ USD 13,858 per QALY) was applied, the probability of cost-effectiveness increased to 73% (Fig. 3b).

**Fig. 4:**
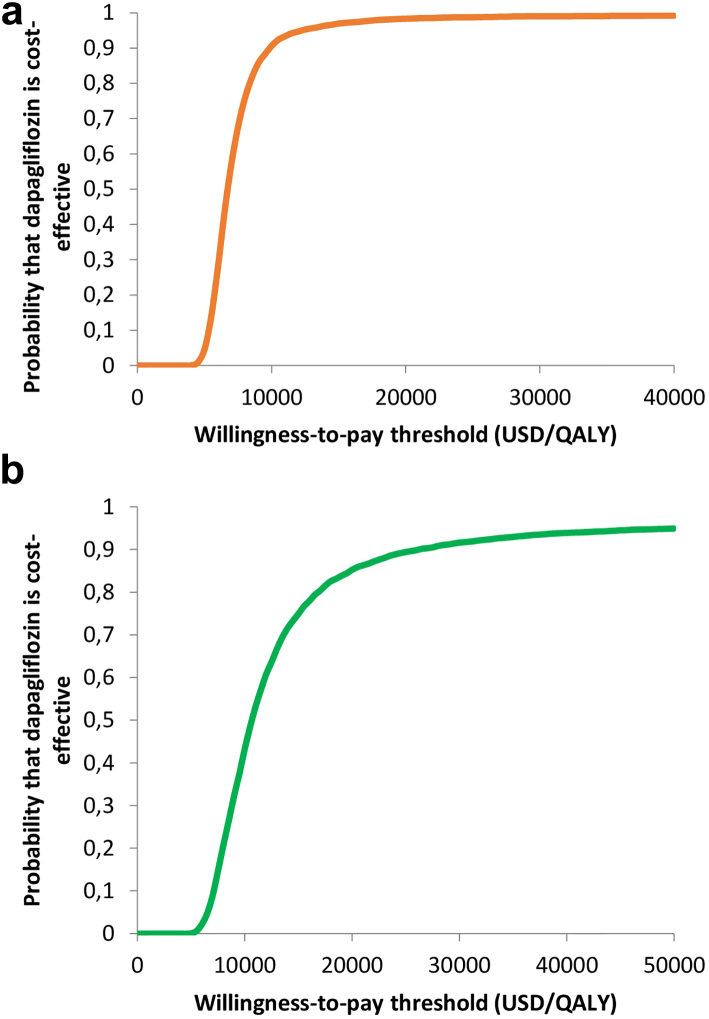
Cost-effectiveness acceptability curves for DAPA-HF (a) and DELIVER (b). The curves depict the probability that dapagliflozin is cost-effective across a range of willingness-to-pay thresholds, based on the probabilistic sensitivity analysis. Abbreviations: QALY, quality-adjusted life-year; USD, United States dollars.

For the DAPA-HF model, the probability that dapagliflozin is cost-effective increases progressively as the willingness-to-pay threshold rises (Fig. 4a). At low thresholds (≤ USD 3000 per QALY), the probability is essentially negligible, increasing markedly from approximately USD 5000 per QALY onward. At thresholds around USD 9000–10,000 per QALY, the probability exceeds 85–90% and approaches 99% at thresholds equal to or above USD 25,000–30,000 per QALY. For the DELIVER model, the probability that dapagliflozin is cost-effective is close to zero at willingness-to-pay thresholds below USD 4500 per QALY and increases progressively beyond this value (Fig. 4b). Around USD 10,000 per QALY, the probability reaches approximately 45–50%, exceeding 70% at thresholds near USD 15,000 per QALY. Beyond USD 25,000 per QALY, the probability stabilizes above 90%, reaching approximately 95% at thresholds of USD 50,000 per QALY.

### Scenario analysis

The ICER decreased consistently with longer time horizons and lower discount rates, and was markedly higher under shorter time horizons, particularly in the DELIVER model (Table 5).

**Table 5:** Scenario analysis.

Scenario	DAPA-HF model (USD/QALY)	DELIVER model (USD/QALY)
Base case	6671.61	10,371.29
Age-related attenuation of excess HF mortality	6506.59	10,313.30
Time horizon		
1 year	49,187.61	111,169.14
5 years	13,245.50	28,114.96
10 years	8699.76	16,437.71
Discount rate		
1%	6347.06	9566.08
5%	7005.75	11,211.05
10%	7869.44	13,413.99
Baseline HF utility		
0.64	9171.81	14,095.10
0.72	8153.32	12,530.23
Dapagliflozin price reduction		
−10%	6159.02	9342.97
−25%	5390.13	7800.48
−50%	4108.65	5229.68

Abbreviations: HF, heart failure; ICER, incremental cost-effectiveness ratio; QALY, quality-adjusted life-year; USD, United States dollars.

## Discussion

In this first economic evaluation conducted in Argentina of dapagliflozin added to standard therapy for chronic HF, we found that the intervention demonstrates a favorable cost-effectiveness profile, with a clear gradient according to ejection fraction. In the DAPA-HF–based model (HF with reduced ejection fraction), dapagliflozin increased QALYs by 0.76 (and life-years by 0.86) at an incremental cost of USD 5069, resulting in an ICER of USD 6672 per QALY, a value compatible with the willingness-to-pay range of 0.5–1 GDP per capita. In the DELIVER-based model (HF with preserved or mildly reduced ejection fraction), the benefit was smaller (0.44 QALYs and 0.50 life-years) with an incremental cost of USD 4542 and an ICER of USD 10,371 per QALY. Although this result remains within the 1 GDP per capita threshold, it is associated with greater uncertainty at more conservative thresholds. Consistent with this, the probabilistic analysis showed a high probability of cost-effectiveness for DAPA-HF (97% at 1 GDP per capita) and a moderate probability for DELIVER (73% at 1 GDP per capita).

Our findings are consistent with evidence summarized in previous systematic reviews of economic evaluations of SGLT2 inhibitors in HF, which describe more consistent cost-effectiveness in HF with reduced ejection fraction and more heterogeneous results in preserved ejection fraction. In particular, cost-utility studies of SGLT2 inhibitors in HF have demonstrated overall economic benefits from the health-system and payer perspectives, but without statistical significance in subgroups with preserved or mildly reduced ejection fraction, suggesting a ceiling of economic uncertainty for this population.^25^

A relevant point of convergence between our evaluation and the international literature is the structure of the main drivers of uncertainty. Systematic reviews of economic evaluations of dapagliflozin and empagliflozin in HF consistently report that results are especially sensitive to drug price and to assumptions related to cardiovascular mortality and HF hospitalizations.^5^^,^^26^ In our model, the ICER was most sensitive to the treatment effect on mortality—particularly cardiovascular mortality—and to the monthly cost of dapagliflozin, followed by baseline utility parameters. In the Argentine context, public procurement strategies and price negotiations are likely the most direct mechanisms to increase the probability of cost-effectiveness, particularly in HF with preserved or mildly reduced ejection fraction, where price discounts of 25–50% can substantially reduce the ICER.

Another relevant consideration when comparing our findings with previous economic evaluations is the variability in QALY estimation across models, even when the same clinical trial evidence informs analyses.^4^^,^^22^ Incremental QALYs may differ depending on the time horizon, discount rate, baseline utility values, utility mapping approach, duration of event-related disutilities, and assumptions regarding post-event recovery. Some economic evaluations based on DAPA-HF and DELIVER have incorporated Kansas City Cardiomyopathy Questionnaire (KCCQ)-based health-state approaches, whereas our analysis used published EQ-5D-based utilities applied to broader Markov health states. In addition, acute heart failure events were modeled as transient one-cycle states, after which patients returned to the stable heart failure state or died. This assumption may represent an optimistic simplification relative to the long-term deterioration in health status frequently observed after heart failure hospitalization in clinical practice and may partially explain why our QALY gains are higher than those reported in some previous models.

This study has limitations. First, extrapolation to a lifetime horizon requires structural assumptions that may affect long-term estimates of costs and health outcomes. Given that scenario analyses demonstrated a strong dependence on the time horizon, this issue was particularly relevant for the DELIVER model. Lifetime extrapolation required assuming that the excess mortality associated with heart failure observed during the trials remained proportional to age-specific background mortality over time. However, evidence from relative-survival cohorts suggests that the relative excess mortality associated with heart failure may attenuate with advancing age, particularly among older populations, although subgroup analyses from DAPA-HF and DELIVER demonstrated broadly consistent treatment effects across age groups.^23^^,^^27^^,^^28^ To explore this uncertainty, we conducted an additional structural scenario analysis assuming progressive attenuation of excess mortality in older age groups. Results remained broadly consistent with the base-case analysis in both models, suggesting that the principal conclusions were robust to this alternative mortality trajectory. Nevertheless, uncertainty surrounding long-term mortality extrapolation remains an inherent limitation of lifetime economic models and should be considered when interpreting the findings. In addition, external validation against the Argentine OFFICE–IC–AR registry supported the overall epidemiological plausibility of the model.^24^ Mortality estimates generated by the model were broadly consistent with those reported in the registry. However, hospitalization rates observed in OFFICE–IC–AR were lower than those projected by the trial-based models. This difference likely reflects the inherent challenges of comparing outcomes derived from randomized clinical trial populations with those observed in routine clinical practice, where patient characteristics, healthcare utilization patterns, and follow-up procedures may differ. Consequently, although the external validation findings provide reassurance regarding the applicability of the model to the Argentine setting, some uncertainty remains regarding the transferability of trial-derived event rates to real-world populations. This limitation should be considered when interpreting the results.

Second, in the DAPA-HF–based model, HF urgent visits could be incorporated only as first events, given the lack of information on recurrences. This simplification may underestimate costs and disutilities associated with repeated events and would therefore bias results conservatively with respect to the incremental benefit of dapagliflozin. Third, reliance on international utility estimates represents a relevant limitation. However, scenario analyses using more conservative utility values allowed us to assess the robustness of results under potential overestimation of baseline quality of life, showing that the cost-effectiveness profile remains favorable in HF with reduced ejection fraction, while uncertainty increases in preserved or mildly reduced ejection fraction. This study has additional limitations related to the explicit consideration of sex, gender, and race or ethnicity. Although DAPA-HF and DELIVER generally demonstrated consistent treatment effects across sex subgroups,^29^ the present economic model was based on aggregate trial estimates and did not incorporate sex-specific treatment effects, healthcare utilization patterns, costs, or outcomes. Furthermore, gender-related factors that may influence access to care, adherence, or disease burden were not incorporated into the analysis. Similarly, potential differences across racial and ethnic groups were not explicitly modeled, and the multinational trial populations may not fully reflect the demographic and social characteristics of patients with heart failure in Argentina. These factors may limit the generalisability of the findings, particularly in real-world settings where social and structural determinants of health play an important role. Future research incorporating sex-disaggregated data and more detailed analyses of demographic and equity-related factors would help to better understand the distribution of benefits and costs across different population groups. Finally, although clinical trials have not demonstrated statistically significant differences in overall adverse event rates with the addition of dapagliflozin to standard therapy, the lack of explicit incorporation of adverse events in the model could lead to a slight underestimation of marginal costs and disutilities associated with treatment in real-world clinical practice.

Dapagliflozin added to standard therapy is cost-effective in Argentina, with greater robustness in HF with reduced ejection fraction. In HF with preserved or mildly reduced ejection fraction, the cost-effectiveness profile is compatible with a threshold of 1 GDP per capita, although with greater uncertainty at more conservative thresholds; price negotiation strategies could increase the probability of cost-effectiveness.

## Contributors

JSA conceived the study and led the overall project. JSA, FM, and DSN designed the economic model and performed the analysis. DSN, FM, GAJ, SIL, JAS, MCP, and JAZ contributed to data interpretation, validation of clinical assumptions, and provided input on local clinical practice and resource use. JSA drafted the manuscript. All authors critically revised the manuscript for important intellectual content, had full access to the data, and approved the final version for submission. JSA, DSN, and FM directly accessed and verified the underlying data reported in the study.

## Data sharing statement

No individual participant data were collected for this study. The economic model was developed using aggregate data extracted from published clinical trials and publicly available secondary sources. The economic model (Microsoft Excel), input parameter tables, and results of scenario and sensitivity analyses are available from the corresponding author upon reasonable request.

## Use of AI

ChatGPT (OpenAI, GPT-4–based model) was used exclusively to assist with language editing and improvement of readability. The tool was not used for data analysis, data interpretation, generation of results, or verification of references. All outputs were critically reviewed, edited, and validated by the authors, who take full responsibility for the final content of the manuscript.

## Declaration of interests

All authors have completed the ICMJE uniform disclosure form. The authors declare no competing interests related to this work.
