# Supplementary material for: Economic evaluation of dapagliflozin added to standard therapy in chronic heart failure in Argentina: a cost-utility analysis
Source: Lancet Reg Health Am. 2026 Jul 15;62:101571. doi: 10.1016/j.lana.2026.101571 (PMC13382610; doi:10.1016/j.lana.2026.101571)
Supplement: Supplementary Tables [file mmc1.docx]

**Supplementary Material**

**Economic evaluation of dapagliflozin added to standard therapy in chronic heart failure in Argentina: a cost-utility analysis**

**Table of Contents**

| **Item** | **Page** |
| --- | --- |
| Supplementary Table 1. CHEERS 2022 checklist | 2,3 |
| Supplementary Table 2. Cost of a heart failure urgent visit | 4 |
| Supplementary Table 3. Cost of chronic heart failure | 5 |
| Supplementary Table 4. Pharmacological costs for the treatment of chronic heart failure | 6 |

|  |  |
| --- | --- |

| Supplementary Table 1. CHEERS 2022 Reporting Checklist | | |
| --- | --- | --- |
| Item | Reported in section | Page/s |
| TITLE |  |  |
| Title | Identify the study as an economic evaluation and specify the interventions being compared. | 1 |
| ABSTRACT |  |  |
| Abstract | Provide a structured summary that includes background, objectives, perspective, setting, methods (including study design and analytic approach), results, and conclusions. | 2 |
| INTRODUCTION |  |  |
| Background and objectives | Provide the study context, research question, and its practical relevance for decision making in health policy or clinical practice. | 4 |
| METHODS |  |  |
| Health economic analysis plan | Indicate whether a health economic analysis plan was developed and where it is available, if applicable. | NA |
| Study population | Describe the characteristics of the study population (e.g., age range, demographic, socioeconomic, or clinical characteristics). | 5 |
| Setting and location | Provide relevant contextual information that may influence the results. | 4,5 |
| Comparators | Describe the interventions or strategies being compared and justify why they were chosen. | 4-5 |
| Perspective | State the perspective(s) adopted in the study and justify the choice. | 4 |
| Time horizon | State the time horizon(s) for the study and justify their appropriateness. | 4 |
| Discount rate | Report the discount rate(s) used for costs and outcomes and justify the choice. | 8 |
| Selection of outcomes | Describe the outcome measure(s) used to capture benefits and harms. | 4-5 and 6-7 |
| Measurement of outcomes | Describe how outcomes used to capture benefits and harms were measured. | 6,7 |
| Valuation of outcomes | Describe the population and methods used to measure and value outcomes. | 8 |
| Measurement and valuation of resources and costs | Describe how resource use was measured and how costs were valued. | 7-9 |
| Currency, price date, and conversion | Report the dates of the estimated resource quantities and unit costs, and describe the currency and any conversions performed. | 7-9 |
| Rationale and description of the model | If a model was used, describe it in detail and justify its use. Report whether the model is publicly available and where it can be accessed. | 5,6 |
| Analytics and assumptions | Describe methods for analysing or statistically transforming data, extrapolation methods, and approaches for validating any model used. | 9,10 |
| Characterising heterogeneity | Describe methods used to estimate how study results vary across subgroups. | NA |
| Characterising distributional effects | Describe how impacts are distributed across individuals or any adjustments made to reflect priority populations. | NA |
| Characterising uncertainty | Describe methods used to characterise uncertainty in the analysis. | 9,10 |
| Approach to engagement with patients and others affected by the study | Describe any approaches used to involve patients, service recipients, the public, communities, or stakeholders (e.g., clinicians, payers) in the study design. | NA |
| RESULTS |  |  |
| Study parameters | Report all analytic inputs (e.g., values, ranges, references), including assumptions about uncertainty or probability distributions. | 8,9 |
| Summary of main results | Report mean values for the main categories of costs and outcomes and summarise them in the most appropriate overall measure. | 10,11 |
| Effect of uncertainty | Describe how uncertainty in analytic judgments, inputs, or projections affects the results. Report the effect of discount rate and time horizon choices where relevant. | 11-15 |
| Effect of stakeholder engagement | Report any differences stakeholder engagement made to the approach or findings of the study. | NA |
| DISCUSSION |  |  |
| Study findings, limitations, generalisability, and current knowledge | Report key findings, limitations, ethical or equity considerations not captured, and how these may affect patients, policy, or practice. | 16,17 |
| OTHER RELEVANT INFORMATION |  |  |
| Source of funding | Describe how the study was funded and the role of the funder in study identification, design, conduct, and reporting. | 10 |
| Conflicts of interest | Report conflicts of interest in accordance with journal or International Committee of Medical Journal Editors requirements. | 19 |

| Supplementary Table 2. Cost of a heart failure (HF) urgent visit^1^ | | | | | |
| --- | --- | --- | --- | --- | --- |
| Procedure | Use (%) | Frequency (n) | Unit cost (ARS) | Unit cost (USD)^2^ | Expected cost per visit (USD) |
| Emergency department consultation | 100 | 1 | 17,637 | 12.39 | 12.39 |
| Electrocardiogram (ECG) | 100 | 1 | 25,846 | 18.15 | 18.15 |
| Complete blood count | 100 | 1 | 2,650 | 1.86 | 1.86 |
| Aspartate aminotransferase (AST) | 100 | 1 | 1,530 | 1.07 | 1.07 |
| Alanine aminotransferase (ALT) | 100 | 1 | 3,605 | 2.53 | 2.53 |
| Serum urea | 100 | 1 | 1,530 | 1.07 | 1.07 |
| Serum creatinine | 100 | 1 | 1,530 | 1.07 | 1.07 |
| Serum sodium | 100 | 1 | 1,530 | 1.07 | 1.07 |
| Serum potassium | 100 | 1 | 1,530 | 1.07 | 1.07 |
| Serum magnesium | 50 | 1 | 1,530 | 1.07 | 0.54 |
| Blood glucose | 100 | 1 | 1,530 | 1.07 | 1.07 |
| Natriuretic peptide (BNP) | 100 | 1 | 20,000 | 14.05 | 14.05 |
| Urinalysis | 50 | 1 | 1,530 | 1.07 | 0.54 |
| Chest X-ray | 100 | 1 | 18,436 | 12.95 | 12.95 |
| Transthoracic echocardiogram | 20 | 1 | 182,643 | 128.28 | 25.66 |
| Intravenous furosemide (20 mg/ampoule) | 100 | 2 | 878 | 0.62 | 1.23 |
| Troponin | 50 | 1 | 15,915 | 11.18 | 5.59 |

¹ Adapted from Naves MCX et al. (21). Costs were obtained from the most recent purchase order of Hospital de Alta Complejidad Cuenca Alta “Néstor Kirchner”.² USD exchange rate as of December 1, 2025: 1 U.S. dollar = ARS 1,423.76, according to the Central Bank of the Argentine Republic. Abbreviations: ALT, alanine aminotransferase; ARS, Argentine pesos; AST, aspartate aminotransferase; BNP, B-type natriuretic peptide; ECG, electrocardiogram; HF, heart failure; USD, United States dollars.

| Supplementary Table 3. Cost of chronic heart failure^1^ | | | | | |
| --- | --- | --- | --- | --- | --- |
| Procedure | Use (%) | Annual frequency (n) | Unit cost (ARS) | Unit cost (USD)^2^ | Expected annual cost (USD) |
| Cardiology consultation | 100 | 4 | 17,637 | 12.39 | 49.55 |
| Electrocardiogram (ECG) | 100 | 4 | 25,846 | 18.15 | 72.61 |
| Complete blood count | 100 | 4 | 2,650 | 1.86 | 7.45 |
| Aspartate aminotransferase (AST) | 100 | 4 | 1,530 | 1.07 | 4.30 |
| Alanine aminotransferase (ALT) | 100 | 4 | 1,530 | 1.07 | 4.30 |
| Blood urea nitrogen (BUN) | 100 | 4 | 3,605 | 2.53 | 10.13 |
| Serum creatinine | 100 | 4 | 1,530 | 1.07 | 4.30 |
| Serum sodium | 100 | 4 | 1,530 | 1.07 | 4.30 |
| Serum potassium | 100 | 4 | 1,530 | 1.07 | 4.30 |
| Serum calcium | 100 | 4 | 1,530 | 1.07 | 4.30 |
| Serum magnesium | 100 | 4 | 1,530 | 1.07 | 4.30 |
| Blood glucose | 100 | 4 | 1,530 | 1.07 | 4.30 |
| Glycated hemoglobin (HbA1c) | 100 | 4 | 1,530 | 1.07 | 4.30 |
| Total cholesterol | 100 | 1 | 1,530 | 1.07 | 1.07 |
| HDL cholesterol | 100 | 1 | 1,530 | 1.07 | 1.07 |
| LDL cholesterol | 100 | 1 | 1,530 | 1.07 | 1.07 |
| Triglycerides | 100 | 1 | 1,530 | 1.07 | 1.07 |
| Thyroid-stimulating hormone (TSH) | 100 | 1 | 15,915 | 11.18 | 11.18 |
| Serum iron | 100 | 1 | 1,530 | 1.07 | 1.07 |
| Ferritin | 100 | 1 | 15,915 | 11.18 | 11.18 |
| Transferrin saturation | 100 | 1 | 1,530 | 1.07 | 1.07 |
| Urinalysis | 100 | 4 | 1,530 | 1.07 | 4.30 |
| Natriuretic peptide (BNP) | 100 | 1 | 20,000 | 14.05 | 14.05 |
| Transthoracic echocardiogram | 100 | 1 | 182,643 | 128.28 | 128.28 |
| Chest X-ray | 100 | 2 | 18,436 | 12.95 | 25.90 |
| Exercise stress test | 8 | 1 | 25,018 | 17.57 | 17.57 |
| *Trypanosoma cruzi* serology (Chagas disease) | 40 | 1 | 6,366 | 4.47 | 4.47 |
| HIV serology | 40 | 1 | 6,366 | 4.47 | 4.47 |
| Ambulatory blood pressure monitoring | 100 | 1 | 109,189 | 76.69 | 76.69 |
| 24-hour Holter monitoring | 100 | 1 | 65,239 | 45.82 | 45.82 |
| Cardiac rehabilitation³ | 85 | 36 | 14,991 | 10.53 | 379.06 |

¹ Adapted from Naves MCX et al. (21). Costs were obtained from the most recent purchase order of Hospital de Alta Complejidad Cuenca Alta “Néstor Kirchner”. ² USD exchange rate as of December 1, 2025: 1 U.S. dollar = ARS 1,423.76, according to the Central Bank of the Argentine Republic. ³ Applicable only to heart failure with reduced ejection fraction. Abbreviations: ALT, alanine aminotransferase; ARS, Argentine pesos; AST, aspartate aminotransferase; BNP, B-type natriuretic peptide; BUN, blood urea nitrogen; ECG, electrocardiogram; HbA1c, glycated haemoglobin; HDL, high-density lipoprotein; HF, heart failure; HIV, human immunodeficiency virus; LDL, low-density lipoprotein; TSH, thyroid-stimulating hormone; USD, United States dollars.

| Supplementary Table 4. Pharmacological costs for the treatment of chronic heart failure¹ | | | | | |
| --- | --- | --- | --- | --- | --- |
| Drug | Dose and frequency | Annual median (ARS) | Annual median (USD)² | Use (%)³ | Weighted annual cost (USD) |
| DAPA-HF | | | | | |
| Enalapril | 20 mg every 12 h | 277,633 | 195 | 56.0 | 109 |
| Losartan | 100 mg every 12 h | 662,046 | 465 | 27.0 | 125 |
| Sacubitril/valsartan | 200 mg every 12 h | 2,233,900 | 1,569 | 11.0 | 173 |
| Carvedilol | 25 mg every 12 h | 466,990 | 328 | 96.0 | 315 |
| Spironolactone | 25 mg every 24 h | 313,227 | 220 | 71.0 | 156 |
| Furosemide | 40 mg every 12 h | 358,785 | 252 | 95.0 | 239 |
| Digoxin | 0.125 mg every 24 h | 56,950 | 40 | 19.0 | 8 |
| Dapagliflozin | 10 mg every 24 h | 808,704 | 568 | 100 | 568 |
| DELIVER | | | | | |
| Enalapril | 20 mg every 12 h | 277,633 | 195 | 36.5 | 71 |
| Losartan | 100 mg every 12 h | 662,046 | 465 | 36.2 | 168 |
| Sacubitril/valsartan | 200 mg every 12 h | 2,233,900 | 1,569 | 5.3 | 83 |
| Carvedilol | 25 mg every 12 h | 466,990 | 328 | 82.8 | 272 |
| Spironolactone | 25 mg every 24 h | 313,227 | 220 | 42.8 | 94 |
| Furosemide | 40 mg every 12 h | 358,785 | 252 | 76.7 | 193 |
| Dapagliflozin | 10 mg every 24 h | 808,704 | 568 | 100 | 568 |

¹ Costs were obtained from the official Argentine medication price website (<https://www.argentina.gob.ar/precios-de-medicamentos>), accessed on December 1, 2025. ² USD exchange rate as of December 1, 2025: 1 U.S. dollar = ARS 1,423.76, according to the Central Bank of the Argentine Republic. ³ Percentage of use according to each clinical trial (DAPA-HF and DELIVER). Abbreviations: ARS, Argentine pesos; USD, United States dollars.
